# The Interplay between Myc and CTP Synthase in *Drosophila*

**DOI:** 10.1371/journal.pgen.1005867

**Published:** 2016-02-18

**Authors:** Gabriel N. Aughey, Stuart J. Grice, Ji-Long Liu

**Affiliations:** MRC Functional Genomics Unit, Department of Physiology, Anatomy and Genetics, University of Oxford, Oxford, United Kingdom; Emory University School of Medicine, UNITED STATES

## Abstract

CTP synthase (CTPsyn) is essential for the biosynthesis of pyrimidine nucleotides. It has been shown that CTPsyn is incorporated into a novel cytoplasmic structure which has been termed the cytoophidium. Here, we report that Myc regulates cytoophidium formation during *Drosophila* oogenesis. We have found that Myc protein levels correlate with cytoophidium abundance in follicle epithelia. Reducing Myc levels results in cytoophidium loss and small nuclear size in follicle cells, while overexpression of Myc increases the length of cytoophidia and the nuclear size of follicle cells. Ectopic expression of Myc induces cytoophidium formation in late stage follicle cells. Furthermore, knock-down of *CTPsyn* is sufficient to suppress the overgrowth phenotype induced by Myc overexpression, suggesting CTPsyn acts downstream of Myc and is required for Myc-mediated cell size control. Taken together, our data suggest a functional link between Myc, a renowned oncogene, and the essential nucleotide biosynthetic enzyme CTPsyn.

## Author Summary

The coordination of metabolism with cell growth is critical for regulation of organismal development. Therefore there is significant interplay between metabolic enzymes and key developmental regulators such as transcription factors. The enzyme CTP synthase (CTPsyn) is essential for metabolic homeostasis as well as growth and development, due to its role in synthesising precursors for many fundamental cellular macromolecules such as RNA and lipids. However, the mechanisms by which CTPsyn is regulated during development are little understood. Here we have shown that Myc, an oncogene and a key developmental regulator, is necessary and sufficient for the assembly of CTPsyn-containing macrostructures termed cytoophidia. We show that the presence of CTPsyn is required for Myc to mediate its effect on cell growth during *Drosophila* oogenesis. Roles for CTPsyn and Myc in tumourigenesis have been well established and both proteins have been considered promising therapeutic targets. By better understanding the relationship between these two proteins, we can gain important insights, not only into tumour pathology and aetiology, but also metazoan developmental processes.

## Introduction

CTP synthase (CTPsyn) is the rate limiting enzyme of the *de novo* synthesis pathway for the nucleotide cytidine-5’-triphosphate (CTP) [1–5]. We and others have observed that CTPsyn is able to form evolutionarily conserved filamentous structures in diverse organisms including *C*. *crescentus*, *S*. *cerevisiae*, *S*. *pombe* and *Drosophila* as well as mammalian cultured cells [6–11]. These structures have been termed cytoophidia. Recently, it has been demonstrated by independent studies that polymerisation of CTPsyn into cytoplasmic filaments acts to attenuate or activate enzymatic activity in response to various environmental and developmental stimuli [12–15].

The coordination of tissue growth and development requires tight control of cellular homeostasis and metabolism. The production of purine and pyrimidine nucleotides is central to these processes. As the rate-limiting enzyme in pyrimidine synthesis, it is particularly important to understand how CTPsyn is regulated at a transcriptional, translational, and post-translational level. Previously we have shown that reversible compartmentalisation of CTPsyn into cytoophidia is involved in the regulation of developmental processes, neuroblast quiescence and cell cycle re-entry [14]. However, the mechanisms by which cytoophidia assembly and nucleotide metabolism are regulated during developmental processes remain little understood.

**Fig 1:**
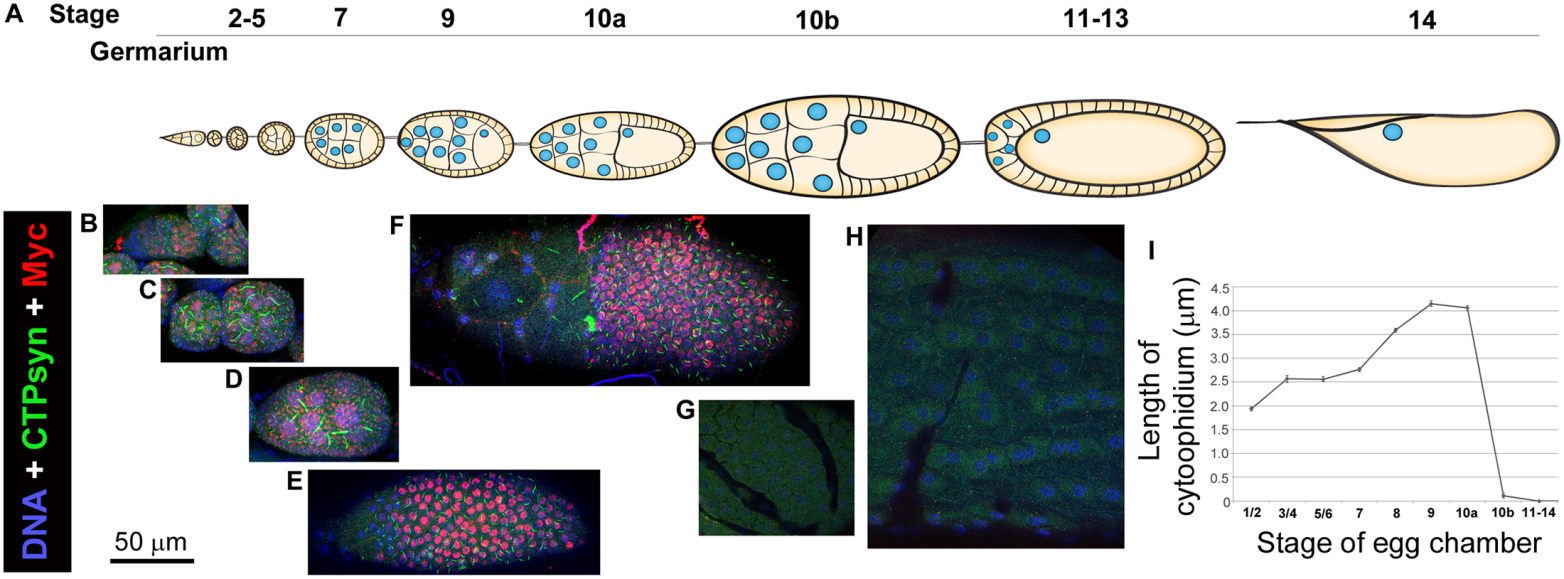
Cytoophidium formation correlates with Myc expression in *Drosophila* follicle cells. (**A**) Schematic representation of the stages of *Drosophila* oogenesis within an ovariole. (**B-H**) Immunostaining of *Drosophila* follicle cells at different stages with antibodies against Myc and CTPsyn. Myc expression is observed at Region 2 of the germarium (**B**) and is continuously expressed at early- (**C**) and mid-oogenesis (**D, E**) until Stage 10a (**F**). Myc expression in follicle cells drops from Stage 10b (**G**) to late-stage egg chambers (**H**). The appearance of cytoophidia correlates with Myc expression. Cytoophidia are detectable from Region 2 to stage 10a (**B-F**). (**B**) Cytoophidia are first observed at region 2 of the germarium concomitant with high Myc expression. (**C**) Two egg chambers at stages 2–5. Note large filamentous structures are macro-cytoophidia in germline cells. (**D**) An egg chamber at Stage 7. Note large filamentous structures are macro-cytoophidia in germline cells. (**E**) Follicle cells of a Stage-9 egg chamber. (**F**) Follicle cells of a Stage-10a egg chamber. (**G**) Follicle cells of a Stage-10b egg chamber. In stage 10b follicle cells, Myc expression is low and cytoophidia are hardly detectable. (**H**) Follicle cells of a Stage-12 egg chamber. In stage-12 follicle cells, Myc expression is low and no cytoophidia are detectable. (**I**) Quantification of follicle cell cytoophidia lengths at various stages of oogenesis. Quantification represents the mean cytoophidium length from > 50 cells in > 3 egg chambers at each stage in *w*^*1118*^ flies. See S1–S7 Figs for individual channels of images shown.

Cytoophidia are consistently observed in several different cell types in *Drosophila* [6,8,9,15,16]. It has been reported that cytoophidia are highly abundant in both the germline nurse cells and the somatic follicle cells of *Drosophila* ovaries [17] (Fig 1A). The follicle cell epithelium provides a particularly attractive system in which to study CTPsyn compartmentalisation, as a single large cytoophidium is present reliably during much of oogenesis. It is unsurprising that CTPsyn is required in large amounts in these tissues as they have a high demand for nucleotides due to their role in synthesising nutrients for the developing oocytes.

The basic-helix-loop-helix transcription factor, Myc, is essential for the regulation of development in *Drosophila* larval and adult tissues [18–24]. Myc is highly expressed in the female germline and is required for generating large polyploid cells through the regulation of endoreplication [22]. To gain a greater understanding of cytoophidia function and regulation, we have characterised the formation of cytoophidia in follicle cells throughout oogenesis. Using *Drosophila* oogenesis as a model system, here we report that Myc regulates cytoophidium formation. We have found that reducing Myc levels results in cytoophidium loss and small nuclear size in follicle cells. Conversely, overexpression of Myc increases the length of cytoophidia and the nuclear size of follicle cells. In addition, we find that cytoophidia can be induced in late stage follicle cells if Myc is ectopically expressed. Furthermore, we show evidence supporting that CTPsyn is required for Myc-mediated cell size control. We conclude that Myc is necessary and sufficient for normal CTPsyn distribution in follicle cells, and that CTPsyn in turn is required for Myc mediated overgrowth.

## Results

### Cytoophidium formation correlates to Myc expression

We previously noted that cytoophidia are consistently observed with a stereotypical distribution in ovarian follicle cells, in which Myc is highly expressed and necessary for cell growth. We decided to investigate the relationship between these two components, initially by further characterising their relative distributions in follicle cells. We stained *Drosophila* egg chambers with two antibodies specifically against *Drosophila* Myc (Fig 1). Both antibodies have revealed that Myc protein levels are high at the germline stem cells and low in cystoblasts at Region 1 (Fig 1B; S1 Fig). This observation is consistent with previous studies [25,26]. The biogenesis of follicle cells starts at Region 2, where follicle stem cells reside. Myc is present at high levels in follicle cells during early- and mid-oogenesis. i.e from Region 2 until Stage 10a (Fig 1C–1F; S2–S5 Figs). Myc levels then drop dramatically at Stage 10b (Fig 1G; S6 Fig) and remain low during late oogenesis (Fig 1H; S7 Fig).

Cytoophidium formation correlates to Myc expression in *Drosophila* follicle cells (Fig 1B–1H). Cytoophidia appear at Region 2 where Myc protein levels also increase. Cytoophidia persist in follicle cells during early- and mid-oogenesis, reaching to 4–5 μm at stage 9 and 10a. Coinciding with the dramatic drop of Myc protein at stage 10b, cytoophidia disappear in the majority of stage-10b follicle cells. Cytoophidia remain undetectable in follicle cells during late oogenesis (i.e. from stage 11 to stage 14). Quantification of cytoophidia length in follicle cells indicates that whilst the length of follicle cell cytoophidia varies throughout oogenesis, cytoophidia length during any particular stage display vary little variation (Fig 1I).

### Myc is required for cytoophidia assembly

**Fig 2:**
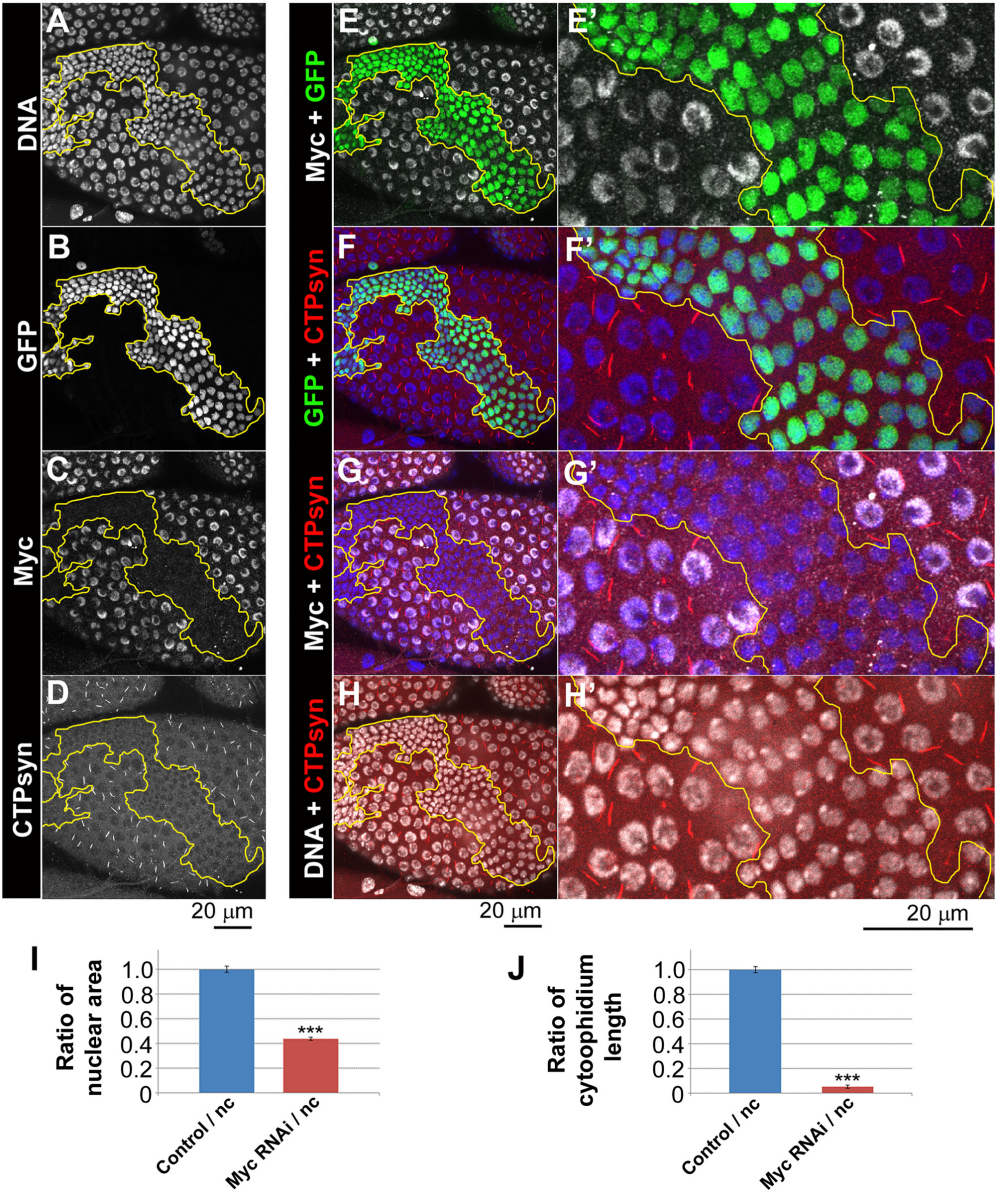
*Myc* knockdown reduces cytoophidium formation in follicle cells. *UAS-Myc-RNAi*^*JF01761*^ clones (i.e. *Myc* RNAi) marked with GFP (**B**, outlined in yellow in **A-H**) have decreased levels of Myc (**C**) and have no detectable cytoophidia as indicated by an antibody against CTP synthase (CTPsyn). DNA staining shows that nuclei of clonal cells (green cells in **E-F**) are smaller than those of neighbouring cells (non-green cells in **E-F**). (**I**) Quantification of nuclear area shows that *Myc* RNAi decreases nuclear size significantly. (**J**) *Myc* RNAi decreases cytoophidium length significantly. ***P<0.001. Error bars show SEM.

It has been reported that the human and mouse orthologues of Myc regulate nucleotide production through expression of nucleotide metabolising enzymes such as CTPsyn in tumour models [27,28]. Having observed that CTPsyn and Myc are both highly expressed in *Drosophila* follicle cells in a similar pattern, we investigated whether Myc is required for cytoophidia assembly. To determine whether Myc has a functional relationship with CTPsyn, *Myc* levels were knocked down using RNAi in clonal populations in the somatic follicle cell epithelium of the *Drosophila* egg chambers using two independent *UAS* driven short hairpin RNAs (shRNA) (Fig 2; S8 Fig). Immunostaining with antibodies against Myc confirmed the efficacy of RNAi mediated Myc knockdown (Fig 2C; S8C Fig). Knockdown of *Myc* resulted in reduced nuclear size (Fig 2A; S8 Fig), consistent with the well-known function of Myc in cell size control. The nuclear area in cells with *Myc* knockdown is less than half of that in non-clonal cells (Fig 2I), which is consistent with what is known about Myc’s role on cell size. Expression of shRNAs targeting *Myc* resulted in a loss of visible cytoophidium formation in GFP marked clones compared to the normal filament formation observed in their neighbours (Fig 2A–2D; S8A–S8E Fig). Quantification showed that the length of residual cytoophidia is less than 5% of the length of cytoophidia in neighbouring cells (Fig 2J). The effect of Myc RNAi on cytoophidium disassembly is not limited to mid-stage egg chambers. In follicle cells at early stage egg chambers, we also observed that Myc RNAi results in cytoophdium disassembly (S9 Fig).

### Myc overexpression induces cytoophidium assembly

**Fig 3:**
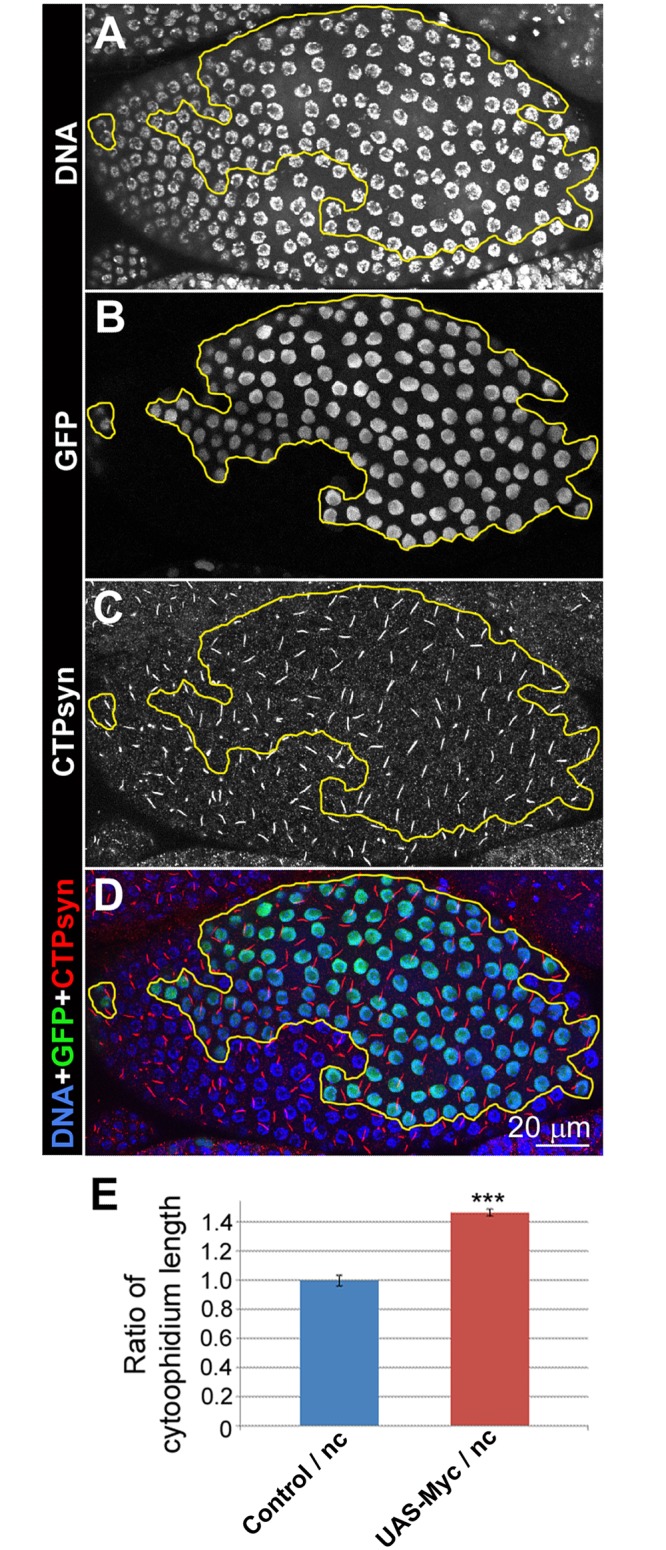
*Myc* overexpression promotes cytoophidium formation in follicle cells. (**A-D**) In stage-8 egg chambers, *UAS*-*Myc* overexpression clones marked with GFP (**B**, outlined in yellow in **A-D**) have longer cytoophidia, as indicated by CTPsyn staining (**C**), than non-GFP cells. DNA staining shows that the nuclei in clones (green cells in **D**) are larger than those of neighbouring cells (non-green cells in **D**). (**E**) Cytoophidia in *Myc* overexpression (*UAS-Myc*) cells increase significantly in length, compared with those in neighbouring cells. ***P<0.001. Error bars show SEM.

**Fig 4:**
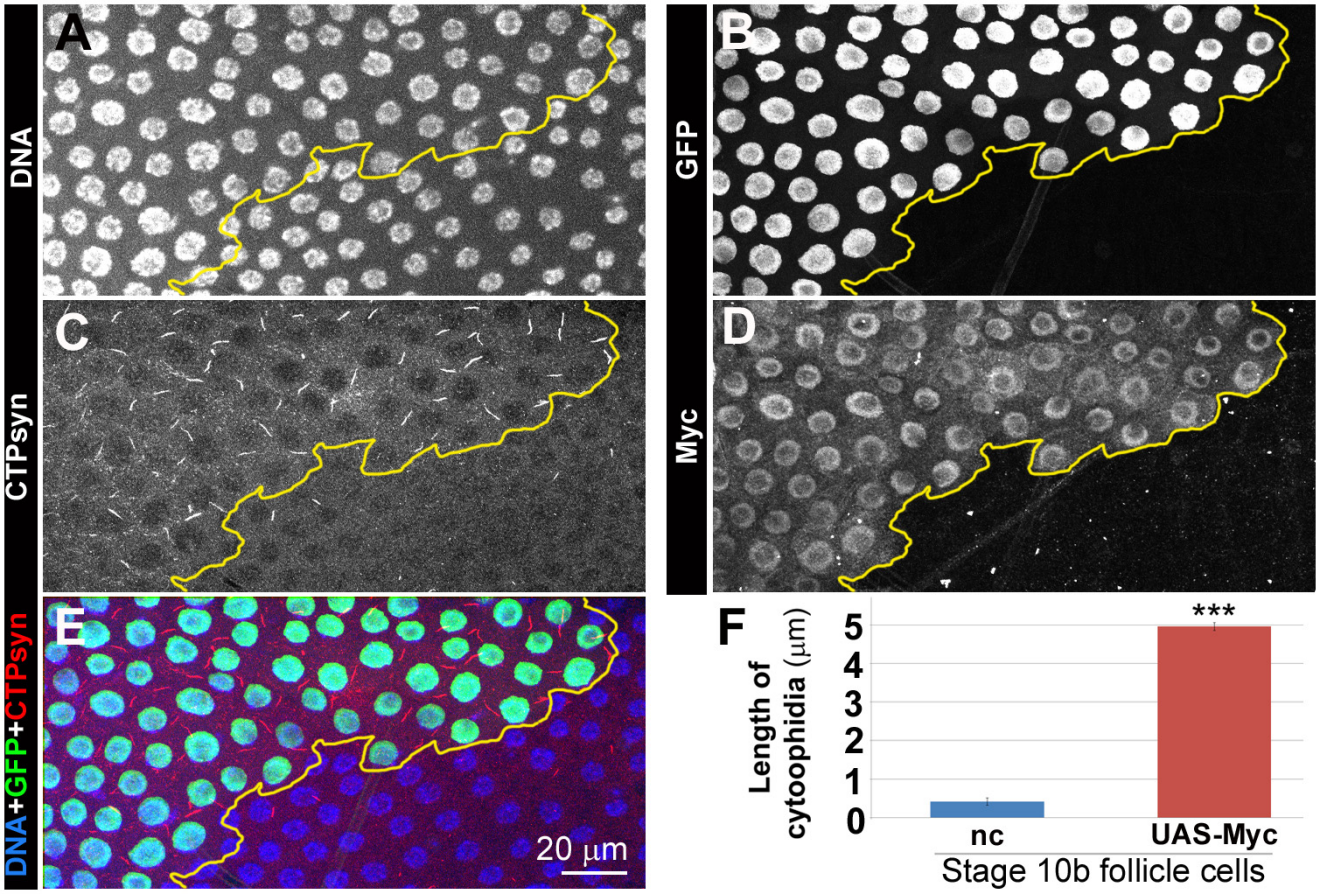
*Myc* overexpression is sufficient to induce cytoophidia formation in Stage 10b follicle cells. (**A-E**) In this stage-10b egg chamber, non-clonal cells (cells that are lack of GFP in B) have low levels of Myc (**D**) and only have very short cytoophidia, as indicated by an antibody against CTPsyn (**C**). *UAS-Myc* overexpression clones (i.e. *Myc* overexpression) marked with GFP (**B**, outlined in yellow in **A-E**) have increased levels of Myc (**D**) and much longer cytoophidia than non-clonal (nc, i.e. non-green) cells (**C**). DNA staining show that the nuclei in clones (green cells in **E**) are larger than those of nc cells (non-green cells in **D**). (**F**) Quantification shows that cytoophidia in *Myc* overexpression (*UAS-Myc*) cells are significantly increased in length, compared to those in nc cells. ***P<0.001. Error bars show SEM.

To further investigate the correlation between Myc levels and CTPsyn, we overexpressed *Myc* in clones in follicle epithelia. During early- and mid-oogenesis, overexpression of *Myc* resulted in increased length of cytooophidia. At stage 8, cytoophidia in follicle cells overexpressing Myc increased about 50% in length compared to those in GFP negative cells (Fig 3). During late oogenesis, cytoophidia are barely detectable in wild-type cells (S6 Fig). However, *Myc* overexpression is sufficient to induce cytoophidium formation in late-stage follicle cells (Fig 4). Overexpression of *Myc* was confirmed by increased level of Myc protein in clonal cells as revealed by immunostaining (Fig 4). Cytoophidia observed in all GFP marked *Myc* overexpression clones in stage 10b can reach 5 μm in length, in contrast to neighbouring cells, which either did not display any cytoophidia, or contained residual cytoophidia with reduced length (Fig 4).

Previous studies have indicated that cytoophidia length can be a function of CTPsyn protein concentration. To determine whether increased cytoophidia prevalence was due to *Myc* mediated upregulation of *CTPsyn* expression, we performed qRT-PCR measurements of *CTPsyn* transcript abundance in *Myc* overexpressing follicle cells, using a 1-hour heat shock of the inducible driver line (S10 Fig). Although the experimental subjects are clonal in nature (with the germline not expressing the Myc transgene), *CTPsyn* expression was significantly upregulated when *Myc* was overexpressed in the follicle cells. Having established that *Myc* overexpression was sufficient to increase *CTPsyn* transcript abundance, we asked whether reduction of *Myc* levels would have a negative effect on *CTPsyn* transcription. qRT-PCR experiments indicated that *CTPsyn* transcript levels were indeed reduced when *Myc* levels were reduced by two RNAi lines (*UAS-Myc-RNAi*^*JF01761*^ and *UAS-Myc-*^*RNAiJF01762*^).

### CTPsyn is required for Myc-dependent cell size control

**Fig 5:**
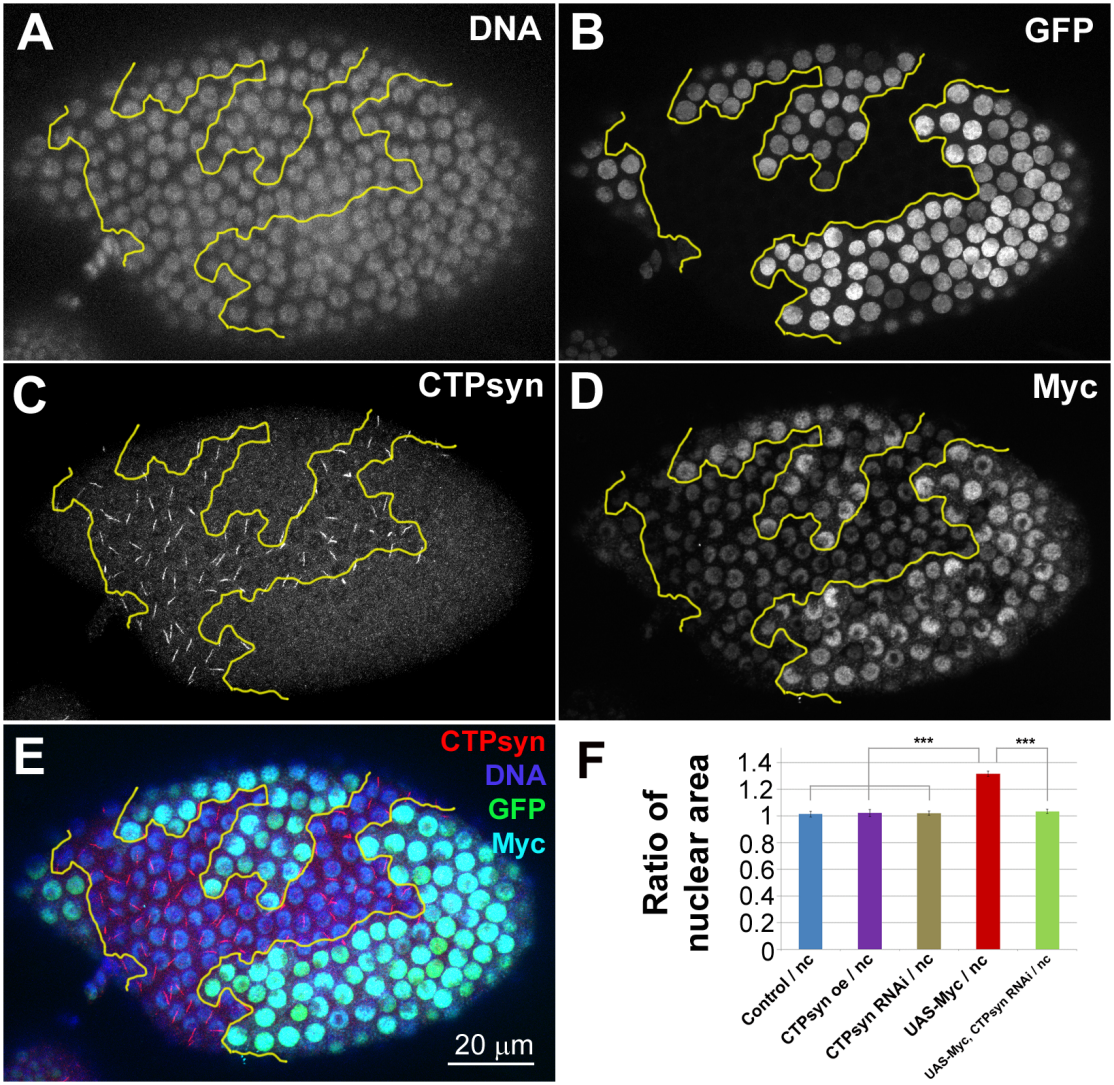
*CTPsyn* knockdown supresses *Myc*-induced overgrowth phenotype in *Drosophila* follicle cells. **(A-E)** The nuclei of cells both overexpressing *Myc* and knocking down *CTPsyn* (*UAS-Myc*, *CTPsyn*^*RNAi*^) are marked by GFP (**B**, outlined by yellow lines in **A-E**). *Myc* overexpression is verified by immunostaining with an antibody against Myc (**D**). *CTPsyn* knockdown is verified by immunostaining with an antibody against CTPsyn (**C**). Note that no cytoophidia are detectable in the clonal cells even when *Myc* is overexpressed. (**F**) Quantification of mid-stage follicle cells shows that *Myc* overexpression (*UAS-Myc*) alone increases nuclear size significantly. Follicle cells in *UAS-Myc*, *CTPsyn*^*RNAi*^ cells have similar nuclear size compared to non-clonal (nc) cells. *CTPsyn*^*RNAi*^ or *CTPsyn* overexpression show no significant difference in nuclear area (see S10 and S11 Figs for representative images). Quantification represents the mean nuclear areas from > 50 cells in > 3 egg chambers per genotype. ***P<0.001. n.s. = not significant. Error bars show SEM.

**Fig 6:**
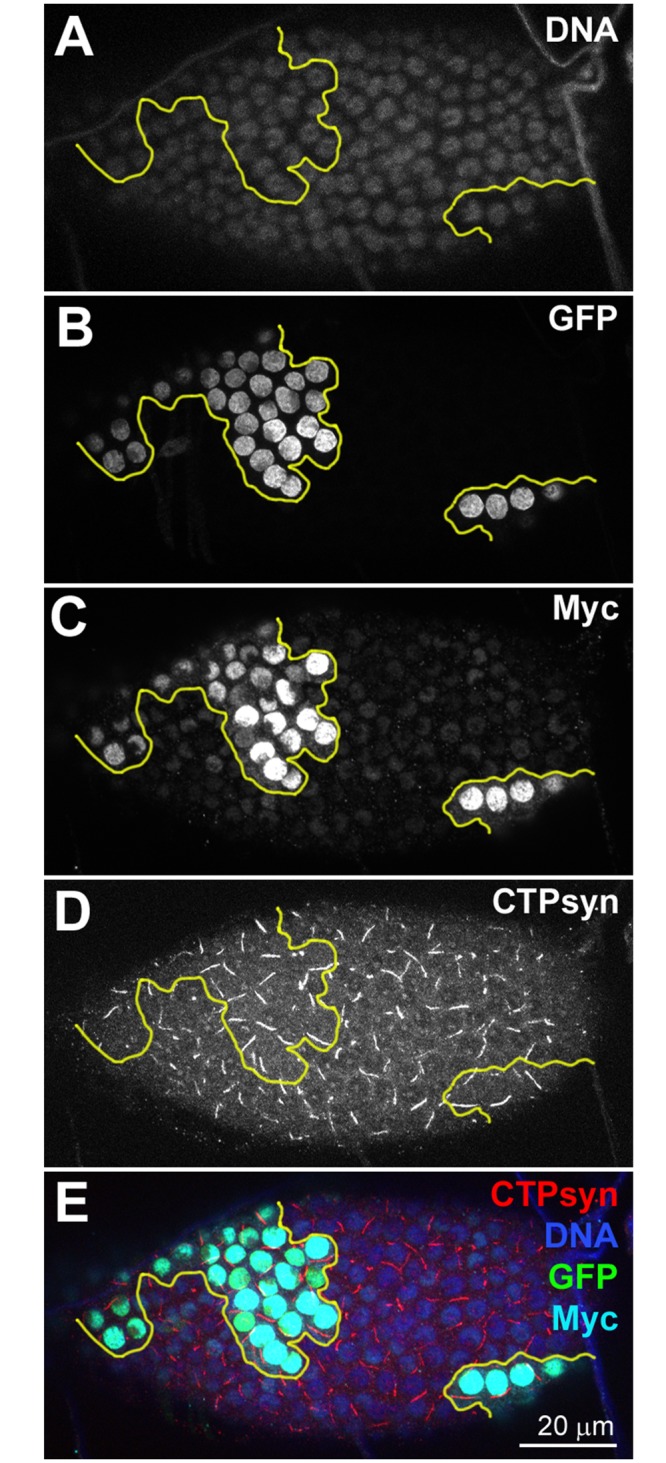
*Myc* overexpression increases cytoophidia length in *Drosophila* follicle cells. **(A-E)** The nuclei of cells overexpressing *Myc* (*UAS-Myc*) are marked by GFP (**B**, outlined by yellow lines in A-E). *Myc* overexpression is verified by immunostaining with an antibody against Myc (**C**). Note that cytoophidia in the clone cells are not only detectable but also longer than those in wild-type cells (comparing those in Fig 5).

Myc has been shown to be a regulator of organ size in *Drosophila*. This regulation occurs partly by regulation of cell size through endocycling [21,22]. When Myc is overexpressed in a clonal population, the overexpressing cells become much larger than their neighbours (Figs 3–6). Given that CTPsyn appears to be acting downstream of Myc signalling we asked whether CTPsyn expression was necessary for Myc dependent control of cell size. To answer this question *CTPsyn* levels were reduced by RNAi in clonal populations of cells also overexpressing *Myc*. Overxpression of *Myc* was confirmed by high levels of Myc protein within GFP labelled clones (Figs 5B, 5D and 6). Ectopic expression of *Myc* led to cytoophidia with increased length (Fig 6B and 6D). When *CTPsyn* was knocked down in cells also overexpressing *Myc*, cytoophidia were no longer detectable indicating effective reduction of *CTPsyn* levels (Fig 5B and 5C, cells marked with GFP). Quantification of nuclear area of cells that overexpressed *Myc*, and knocked-down *CTPsyn* by RNAi (*UAS*-*Myc*, *CTPsyn*^*RNAi*^) revealed that there was a significant decrease in nuclear area compared to *Myc* overexpression alone (Fig 5F). Nuclear size in *UAS-Myc*, *CTPsyn*^RNAi^ cells is comparable to neighbouring cells without CTPsyn^RNAi^ or Myc overexpression. These results indicate that CTPsyn is required for Myc-dependent cell size control.

We also asked whether altering CTPsyn levels alone was sufficient to interfere with follicle cell size control. To determine whether the reduction of CTPsyn alone was sufficient to reduce cell size, we used a previously characterised *CTPsyn* null mutant to generate mitotic clones in follicle cells [16]. Mutant clones showed no difference in cell size compared to adjacent heterozygous or homozygous cells (S11 Fig). Furthermore, *CTPsyn*^RNAi^ clones alone, also showed no difference in nuclear area (S12 Fig). Surprisingly, we could not detect any overt cellular phenotypes in CTPsyn mutant or RNAi cells. Not only was nuclear area unchanged, but nuclear morphology appeared normal (i.e. no pyknosis/karyorrhexis indicating apoptotic cell death).

To determine whether increasing CTPsyn concentration had an effect on nuclear area, we clonally overexpressed Myc in follicle cells. *CTPsyn* overexpressing cells showed no significant difference in nuclear area. We also observed no difference in Myc staining in CTPsyn overexpressing cells (S13 Fig). Together, these data indicate that an interaction between CTPsyn and Myc is necessary for cell size control, whilst altering CTPsyn levels alone has no effect.

## Discussion

The regulation of nucleotide metabolism during metazoan development must be closely controlled for the proper coordination of growth and organogenesis. Interestingly, both the human orthologue of Myc (c-Myc) and CTPsyn have been implicated in tumourigenesis. The proto-oncogene, c-Myc has been previously implicated in mediating aberrant nucleotide synthesis in tumourigenesis [27,28]. c-Myc has been shown to be upregulated in a large number of different tumours and it has been proposed to be involved in as many as 80% of human cancers [29]. Similarly, CTPsyn levels have been shown to frequently be elevated in tumour cells and clustered mutations within the CTPsyn locus in Chinese hamster tumours have been associated with increased proliferative capacity [30].

Myc is central to the regulation of *Drosophila* cell size, cell survival and cell proliferation. It is therefore fundamental for specifying overall tissue size and shape. It is conceivable, therefore, that Myc acts to regulate nucleotide metabolism through CTPsyn, as CTP nucleotides are essential for the synthesis of DNA, RNA and lipids. In a previous study it was noted that several nucleotide metabolising enzymes are upregulated in response to c-Myc expression in a human cancer cell line [28]. Furthermore ChIP-seq experiments have shown that c-Myc binds at the CTPsyn locus in human cells [31]. Several large scale screening studies in fly and human cells have suggested that c-Myc regulates the expression of up to 15% of genes in the genome [32–34]. Although there is evidence to suggest that Myc regulates nucleotide metabolism in tumour models and cell culture, the extent to which this relationship is relevant in normal animal developmental processes, has been less well defined. Our data indicate that Myc regulates pyrimidine synthesis during oogenesis through control of *CTPsyn* expression and cytoophidia abundance.

The control of CTPsyn filamentation via Myc is likely to be complex, with both direct and indirect control mechanisms. Firstly, Myc, a transcription factor that stimulates the expression of all three RNA polymerases [35], may control *CTPsyn* expression at a transcriptional level. In support of this, ChIP-seq experiments have identified c-Myc binding sites at the CTPsyn locus in human cells [31,36]. Furthermore, microarray experiments have shown *CTPsyn* downregulation following *dMyc* knockdown in *Drosophila* cells, with a predicted E-box Myc-binding motif being present in close proximity to the transcriptional start site [37]. Conversely, some studies have reported no changes being detected following *dMyc* knockdown [28,38]. Our data suggest that *CTPsyn* expression is indeed under the control of *Myc* (S10 Fig). Secondly, Myc may also regulate CTPsyn translation. MYC has been shown to directly regulate ribosome biogenesis in *Drosophila*, and this may have an impact on CTPsyn translation. Both ribosome protein/RNA [39] and CTPsyn upregulation are seen to be a common feature of a number of MYC dependent cancers, while CTPsyn overexpression has been shown to increase filament abundance and size [14,16].

In addition to direct transcriptional and translational control, Myc may also control filamentation of CTPsyn on a metabolic level. Myc has been shown to control both glycolytic and glutaminolytic processes that support the energy needs of the cell. However, both glucose and glutamine (a substrate of CTPsyn) are also required for nucleotide biosynthesis, so their use as both anabolic and catabolic substrates must be regulated to coincide with the changing demands of the cell [40]. Nutritional stress has been shown to result in cytoophidia formation, whilst the re-addition of key nutrients promotes the subsequent dissociation of the enzyme from the filament. In addition, the inactivation of the Ser/Thr kinase Akt also modifies filament formation in neural stem cells [14]. Akt is a key kinase that both activates and deactivates metabolic proteins in response to changing nutrient availabilities [41]. Changes in Akt activity, in response to changing nutrients or mitogens, have also been shown to increase Myc translation [42]. Furthermore, Myc upregulation has also been shown to increase levels of phospho-Akt in *Drosophila* [43]. CTPsyn filamentation has been shown to control enzymatic activity [13,14], and CTPsyn stored in the filament may act as a rapidly accessible pool of enzyme that can be used by the cell in response to nutritional changes. How filamentation, and thus enzyme inactivation, is used to help the cell switch between different metabolic programs is now a pertinent direction of inquiry. It is also therefore imperative that the study of how Myc, along with other convergent and differentially regulated factors, regulate CTPsyn filamentation on the post-translational level to maintain metabolic homeostasis.

The observation that cells overexpressing Myc are sensitive to changes in CTPsyn levels, whilst cells with endogenous Myc levels are not, has important implications for tumour biology. Our data suggests that cells with abnormal Myc activity may be more affected by CTPsyn inhibition than cells with normal Myc activity. Determining whether this is also the case in tumour cells could yield therapeutic benefits. It will be interesting to see in future studies whether CTPsyn has synthetic lethal properties in Myc-dependent tumours.

Interestingly, our data indicate that whilst reducing CTPsyn levels in tissues where Myc is overexpressed is sufficient to rescue the Myc dependent cell size phenotype, CTPsyn knockdown in follicle cells had no obvious effect. This is surprising due to the essential role of CTPsyn in the synthesis of pyrimidine nucleotides, and our previous observations noting that *CTPsyn* null mutants are lethal at an early stage [16]. Our observations were restricted to narrow time intervals, so it is possible that more noticeable effects may be seen later in the development of mutant clones. Furthermore, it is possible that these cells are able to continue growing normally due to non cell-autonomous contribution of nucleotides from neighbouring cells in the follicle epithelium. It has been shown previously that diffusion may occur through ring canals (cytoplasmic bridges between adjacent cells) [44]. Diffusion of CTP through the ring canals may be sufficient to supply follicle cells with the required nucleotides for normal cell growth. However, further study is necessary to identify subtle phenotypes resulting from lowered CTPsyn levels.

Previous studies have shown that sequestration of CTPsyn into cytoophidia can downregulate CTPsyn enzymatic activity [12–14]. We showed that this process is involved in developmentally regulated changes in metabolism [14]. Our observation that loss of cytoophidia does not result in severe cellular phenotypes is consistent with these previous observations. Our previous data, together with the evidence presented here, suggests that although large amounts of CTPsyn are present within the follicle cells, the majority is inactive and compartmentalised into cytoophidia throughout much of oogenesis. There is a dramatic reduction in observable cytoophidia during stage 10a-b follicle cells (Fig 1I). This period corresponds to the rapid growth and start of chorion gene amplification [45]. We speculate that previously inactive CTPsyn becomes diffuse and enzymatically active to facilitate growth and rapid chorion gene amplification by upregulating nucleotide production. Further study will be required to determine the cellular stimuli which initiate this change in CTPsyn localisation.

That observation that upregulation of Myc signalling promotes cytoophidia formation whilst increasing cell growth, initially seems paradoxical due to the fact that CTPsyn enzymatic activity is downregulated by filament formation. However, we previously showed that filamentous and diffuse CTPsyn exist in equilibrium, with more enzyme sequestered as protein concentration increases. It was shown that as CTPsyn concentration increases, both diffuse CTPsyn concentration and cytoophidia length increase. However, the ratio of free CTPsyn to filamentous increases only modestly as protein concentration is increased, thereby buffering intracellular CTP pools within narrow limits (within around three-fold) [13,14]. Therefore, it seems that cellular overgrowth driven by Myc overexpression results in a relatively small increase in active CTPsyn, which is nonetheless required for increased cellular growth. It is likely that in Myc overexpressing cells, surplus CTPsyn continues to be sequestered in cytoophidia due to toxicity of CTP concentrations in excess of the constraints imposed by filament formation.

In conclusion, our data describe the interplay between Myc and the essential pyrimidine biosynthesis enzyme, CTPsyn, in the regulation of *Drosophila* tissue development. These results suggest that Myc regulates the coordination of cellular growth and metabolic regulation through transcriptional control of nucleotide biosynthetic enzyme expression and filament formation. Furthermore, this study may help to provide fresh insights into the aetiology of cancers by establishing a connection between two major factors implicated in tumourigenesis.

## Materials and Methods

### *Drosophila* husbandry

All stocks were maintained at 25°C on standard *Drosophila* medium (80g/l maize, 18g/l dried yeast, 10g/l soya flour, 80g/l malt extract, 40g/l molasses, 8g/l agar, 6.6ml acid mix). *w*^*1118*^ (Bloomington stock centre) was used as a wild-type control unless stated otherwise. All RNAi stocks were from the TRiP collection (Bloomington stock centre).

### Immunofluorescence and microscopy

For ovary dissections, flies were fed with wet yeast for at least one day before dissection to ensure reasonable numbers of egg chambers at all stages. All tissues were dissected into Grace’s Insect Medium, fixed in 4% paraformaldehyde in PBS for 10 minutes and washed with PBT (1X PBS + 0.5% horse serum + 0.3% Triton X-100). Tissues were incubated in primary antibodies at room temperature overnight, washed with PBT then incubated at room temperature overnight in secondary antibodies. Primary antibodies used were rabbit anti-CTPsyn (Santa Cruz 134457), goat anti CTPsyn (Santa Cruz 33304), rabbit anti *Drosophila* Myc (d1-717, Santa Cruz 28207) and rabbit anti *Drosophila* MycN (d46-507, Santa Cruz 28208). Secondary antibodies used were: donkey anti-rabbit Cy5 (Jackson 711-175-152), donkey anti-goat Cy5 (Molecular Probes A11055), donkey anti-goat 549 (Jackson 711-585-152). Hoescht 33342 (1μg/ml) was used to label DNA. All samples were imaged using a Leica SP5II confocal microscope.

### “Flip-out” clone generation

All UAS “flip-out” clones were generated using the inducible driver: *HsFLP*, *UAS-GFP*_*nls*_*; UAS-Dcr2; tub>GAL80>GAL4 / SM5*, *Cyo-TM6*, *Tb*. Virgin females of this genotype were crossed to males with UAS-shRNA or UAS-ORFs for clonal knockdown or overexpression respectively. Males from the following genotypes have been used in this study:

1. w[1118]; P{w[+mC] = UAS-Myc.Z}[34]
2. y[1] v[1]; P{y[+t7.7] v[+t1.8] = TRiP.JF01761}attP2
3. y[1] v[1]; P{y[+t7.7] v[+t1.8] = TRiP.JF01762}attP2
4. *UAS-Myc*, *CTPsyn RNAi;* and
5. *w*^*1118*^ as the control group.

Progeny were heat shocked at 96 hours after egg deposition for follicle cell clones. For follicle cell clones female flies without curly wings were selected after eclosing and fed with wet yeast for 24 hours before dissecting. Gal4 expressing clones were identified by presence of nuclear GFP marker.

### Mitotic clone generation

*CTPsyn* mitotic clones were generated using FLP induced mitotic recombination as previously detailed [46]. *w HsFlp; Ubi-GFP FRT2A* virgin females were crossed to w; *CTPsyn*^*d06996*^ FRT2A / Cyo males to generate heterozygous progeny in which recombination could occur. Larvae were heat shocked at 37°C for 45 minutes at 96 hours after egg deposition (AED) to produce follicle cell clones. Upon eclosing, non-curly flies were selected and fed wet yeast for 24 hours before dissection. Mitotic clones were identified by absence of GFP.

### Quantification of images and statistical analysis

Image processing and analysis was conducted using Leica Application Suite Advanced Fluorescence Lite and ImageJ. Nuclear areas are expressed as a ratio of the average nuclear area in GFP marked clones to neighbouring cells (GFP negative). For each genotype over 50 cells were quantified from at least three egg chambers derived from separate animals. ANOVA was performed to check for significant differences between data groups. Significant differences were attributed for p<0.05.

### RNA quantification

Total RNA was prepared from cells using the miRNeasy kit (Qiagen) as per manufacturer’s instructions. 1 μg of RNA was used for reverse transcription using the QuantiTect Reverse Transcription Kit (Qiagen). For qRT-PCR, the cDNA was diluted 1:10, mixed with primers and SYBRGreen Jumpstart Taq readymix (Sigma) and amplified using Applied Biosystems Fast Real-Time PCR platform, using the primers: for CTPsyn, GAGTGATTGCCTCCTCGTTC and TCCAAAAACCGTTCATAGTT; and for *rp49*, GCTAAGCTGTCGCACAAA and GAACTTCTTGAATCCGGTG. The Ct values for *CTPsyn* were normalised with *rp49*, and ΔΔCt values were calculated. The data was calculated using at least two independent biological replicates, each of which utilised three technical replicates.

## Supporting Information

S1 Fig Cytoophidium formation correlates with Myc in the germarium of *Drosophila* ovarioles. (**A**) DNA stained by Hoechst 33342. (**B**) Immunostaining with an antibody against Myc. (**C**) Immunostaining with an antibody against CTPsyn. (**D**) A merge image of **A-C**. Note that Myc levels are high in germline stem cells (indicated by triangles) and drop in cystoblasts in region 1 (**B**). Myc returns to high level at Region 2, from which the biogenesis of follicle cells starts. CTPsyn forms cytoophidia starting at Region 2 (**C**). Arrows mark the boundary between Region 1 and Region 2 in the germarium (outlined by dotted lines).
(TIF)

S2 Fig Cytoophidium formation correlates with Myc expression during *Drosophila* early oogenesis. (**A**) DNA stained by Hoechst 33342. (**B**) Immunostaining with an antibody against Myc. (**C**) Immunostaining with an antibody against CTP synthase (CTPsyn). (**D**) A merge image of **A-C**. In these two egg chambers at stages 2–5, CTPsyn forms small cytoophidia in follicle cells and large cytoophidia in germline cells (**C**). Myc is distributed in the nucleus of follicle cells and germline cells (**B**).
(TIF)

S3 Fig Cytoophidium formation correlates with Myc expression during *Drosophila* mid-oogenesis. (**A**) DNA stained by Hoechst 33342. (**B**) Immunostaining with an antibody against Myc. (**C**) Immunostaining with an antibody against CTPsyn. (**D**) A merge image of **A-C**. In this stage-7 egg chamber, CTPsyn forms small cytoophidia in follicle cells and large cytoophidia in germline cells (**C**). Myc is distributed in the nucleus of follicle cells and germline cells (**B**).
(TIF)

S4 Fig Cytoophidium formation correlates with Myc expression in *Drosophila* follicle cells at stage 9. (**A**) DNA stained by Hoechst 33342. (**B**) Immunostaining with an antibody against Myc. (**C**) Immunostaining with an antibody against CTP synthase (CTPsyn). (**D**) A merge image of **A-C**. In the follicle cells of stage-9 egg chamber, cytoophidia are longer than those at early stages. Myc is distributed in the nucleus of follicle cells (**B**). Note that the images are a surface view of the egg chamber focusing on follicle cells only.
(TIF)

S5 Fig Cytoophidium formation correlates with Myc expression in *Drosophila* follicle cells at stage 10a. (**A**) DNA stained by Hoechst 33342. (**B**) Immunostaining with an antibody against Myc. (**C**) Immunostaining with an antibody against CTPsyn. (**D**) A merge image of **A-C**. Cytoophidia remain detectable in the follicle cells of stage-10a egg chamber. Myc is distributed in the nucleus of follicle cells (**B**). Note that the images are a surface view of the egg chamber focusing on follicle cells only.
(TIF)

S6 Fig Myc expression decreases and cytoophidia disappear in *Drosophila* follicle cells at stage 10b. (**A**) DNA stained by Hoechst 33342. (**B**) Immunostaining with an antibody against Myc. (**C**) Immunostaining with an antibody against CTPsyn. (**D**) A merge image of **A-C**. In the follicle cells of stage-10b egg chamber, immunostaining of Myc shows low signal and cytoophidia are hardly detectable. Note that the images are a surface view of the egg chamber focusing on follicle cells only.
(TIF)

S7 Fig Myc expression decreases and cytoophidia disappear in *Drosophila* follicle cells at stage 12. (**A**) DNA stained by Hoechst 33342. (**B**) Immunostaining with an antibody against Myc. (**C**) Immunostaining with an antibody against CTPsyn. (**D**) A merge image of **A-C**. In the follicle cells of stage-12 egg chamber, immunostaining of Myc shows low signal and cytoophidia are undetectable. Note that the images are a surface view of the egg chamber focusing on follicle cells only.
(TIF)

S8 Fig Knockdown *of Myc* using a second RNAi also reduces cytoophidium formation in follicle cells. *UAS-Myc-RNAi*^*JF01762*^ clones marked with GFP (**B**, outlined in yellow in **A-E**) have decreased levels of Myc (**C**) and have no detectable cytoophidia as indicated by an antibody against CTPsyn (**D**). DNA staining shows that nuclei of GFP cells are smaller than those of neighbouring cells (**A**).
(TIF)

S9 Fig *Myc* knockdown disrupts follicle cell cytoophidia in early stage follicle cells. (**A**) DNA stained by Hoechst 33342. Characteristic small nuclear size can be seen in *Myc-RNAi* cells (yellow outline). (**B**) GFP marked cells indicating expression of *Myc-RNAi* (**C**) Immunostaining with an antibody against CTPsyn. (**D**) Immunostaining with an antibody against Myc. Myc levels are reduced in *Myc-RNAi* cells. (**E**) Merge of **A-D**. (**F**) Merge of **C** and **D**.
(TIF)

S10 Fig Myc regulates *CTPsyn* expression. qRT-PCR was performed to detect Myc dependent changes in *CTPsyn* expression. The levels of *CTPsyn* were analysed in egg chambers overexpressing *CTPsyn* (*UAS-CTPsyn*; positive control), overexpressing *Myc* (*UAS-Myc;* to produce Myc level increases), two Myc RNAi lines (1, *UAS-Myc-RNAi*^*JF01761*^ and 2, *UAS-Myc-RNAi*^*JF01762*^, to produce *Myc* level decreases), and a control with no expression alterations. *CTPsyn* levels were seen to be positively correlated with *Myc* expression. Expression levels were normalised using *rp49*. Error bars show SEM. ANOVA was performed for significance analysis (*P<0.05, **P<0.01, ***P<0.001).
(TIF)

S11 Fig *CTPsyn* knockout does not cause an overgrowth phenotype in *Drosophila* follicle cells. **(A-B)** Mitotic clones of the *CTPsyn*^*d06996*^ mutant. **B** shows a zoom of the region of follicle cells depicted by the box in **A,** panel 4. Mutant cells, that do not express GFP, contain no observable CTPsyn filament (**C-E**) The nuclei area of both wild type (GFP +ive) and *CTPsyn*^*d06996*^ mutant (GFP-ive) cells were measured. The analysis was performed throughout the wild type and mutant regions. (**C**, separated by the white line, quantified in D) and at the boundary of the clones (**C**, example wild-type boundary cell, ‘B’, and mutant cell ‘b’, boundary cells; quantified in **E**). (**D, E**) No differences were observed between wild type and *CTPsyn*^*d06996*^ mutant cell nuclear area. Error bars show SEM.
(TIF)

S12 Fig CTP synthase RNAi affects neither Myc expression nor nuclear size in follicle cells. (**A**) DNA stained by Hoechst 33342. (**B**) GFP marked cells indicating *CTPsyn-RNAi* (yellow outline). (**C**) Immunostaining with an antibody against CTPsyn showing cytoophidia are not detectable in CTPsyn-RNAi cells (green cells in **E**). (**D**) Immunostaining with an antibody against Myc. Myc levels are unchanged in CTPsyn-RNAi cells, comparing with those in non-clonal cells. (**E**) Merge of **A-D**. (**F**) Merge of **C** and **D**.
(TIF)

S13 Fig CTP synthase overexpression affects neither Myc expression nor nuclear size in follicle cells. (**A**) DNA stained by Hoechst 33342. (**B**) GFP marked cells indicating *CTPsyn* overexpression (yellow outline). (**C**) Immunostaining with an antibody against CTPsyn showing cytoophidia increase in length and thickness in clonal cells (green cells in **E**), comparing to non-clonal cells. (**D**) Immunostaining with an antibody against Myc. Myc levels are unchanged in CTPsyn overexpressing cells, comparing with those in non-clonal cells. (**E**) Merge of **A-D**. (**F**) Merge of **C** and **D**.
(TIF)
