# Supplementary figures and images for: The Interplay between Myc and CTP Synthase in Drosophila
Source: PLoS Genet. 2016 Feb 18;12(2):e1005867. doi: 10.1371/journal.pgen.1005867 (PMC4759343; doi:10.1371/journal.pgen.1005867)

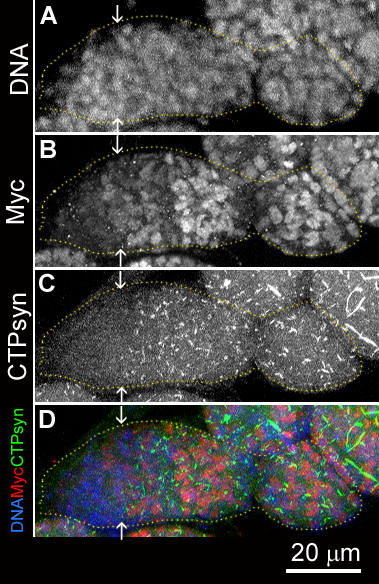

Supplement: S1 Fig — (A) DNA stained by Hoechst 33342. (B) Immunostaining with an antibody against Myc. (C) Immunostaining with an antibody against CTPsyn. (D) A merge image of A-C. Note that Myc levels are high in germline stem cells (indicated by triangles) and drop in cystoblasts in region 1 (B). Myc returns to high level at Region 2, from which the biogenesis of follicle cells starts. CTPsyn forms cytoophidia starting at Region 2 (C). Arrows mark the boundary between Region 1 and Region 2 in the germarium (outlined by dotted lines). (TIF) [file pgen.1005867.s001.tif]

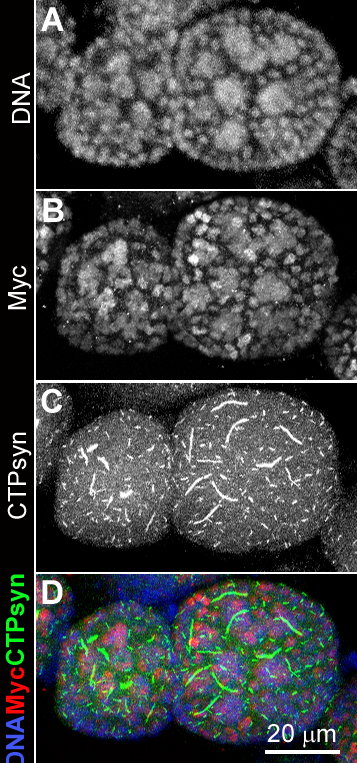

Supplement: S2 Fig — (A) DNA stained by Hoechst 33342. (B) Immunostaining with an antibody against Myc. (C) Immunostaining with an antibody against CTP synthase (CTPsyn). (D) A merge image of A-C. In these two egg chambers at stages 2–5, CTPsyn forms small cytoophidia in follicle cells and large cytoophidia in germline cells (C). Myc is distributed in the nucleus of follicle cells and germline cells (B). (TIF) [file pgen.1005867.s002.tif]

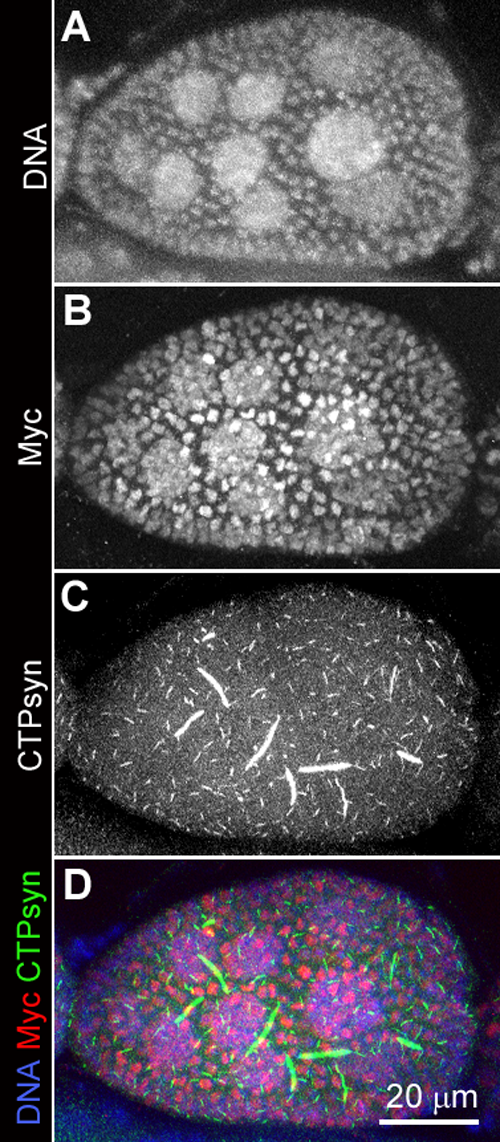

Supplement: S3 Fig — (A) DNA stained by Hoechst 33342. (B) Immunostaining with an antibody against Myc. (C) Immunostaining with an antibody against CTPsyn. (D) A merge image of A-C. In this stage-7 egg chamber, CTPsyn forms small cytoophidia in follicle cells and large cytoophidia in germline cells (C). Myc is distributed in the nucleus of follicle cells and germline cells (B). (TIF) [file pgen.1005867.s003.tif]

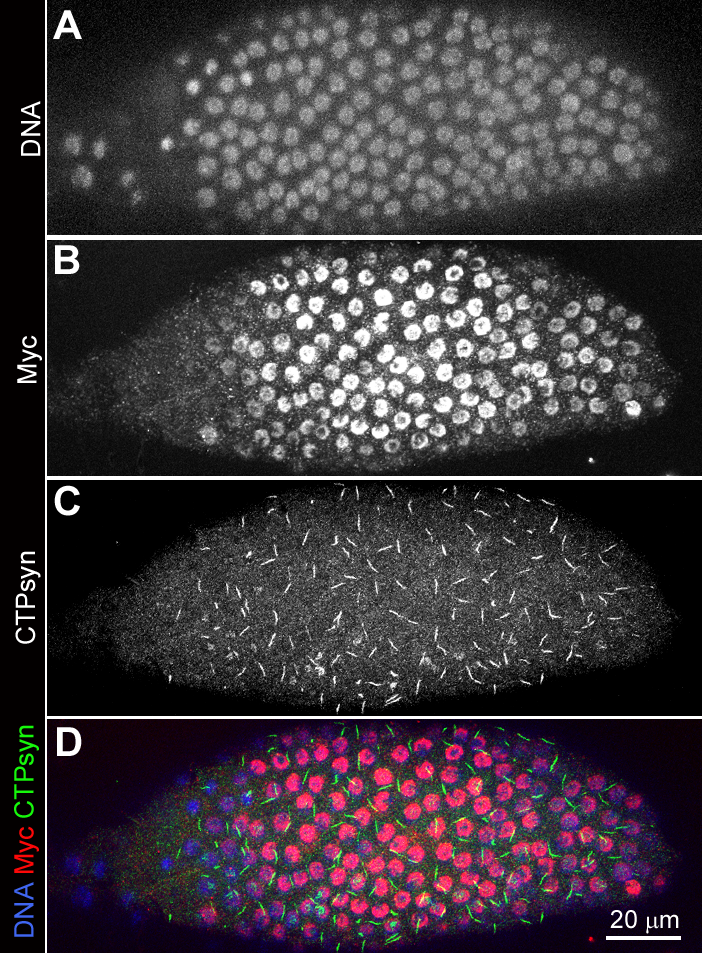

Supplement: S4 Fig — (A) DNA stained by Hoechst 33342. (B) Immunostaining with an antibody against Myc. (C) Immunostaining with an antibody against CTP synthase (CTPsyn). (D) A merge image of A-C. In the follicle cells of stage-9 egg chamber, cytoophidia are longer than those at early stages. Myc is distributed in the nucleus of follicle cells (B). Note that the images are a surface view of the egg chamber focusing on follicle cells only. (TIF) [file pgen.1005867.s004.tif]

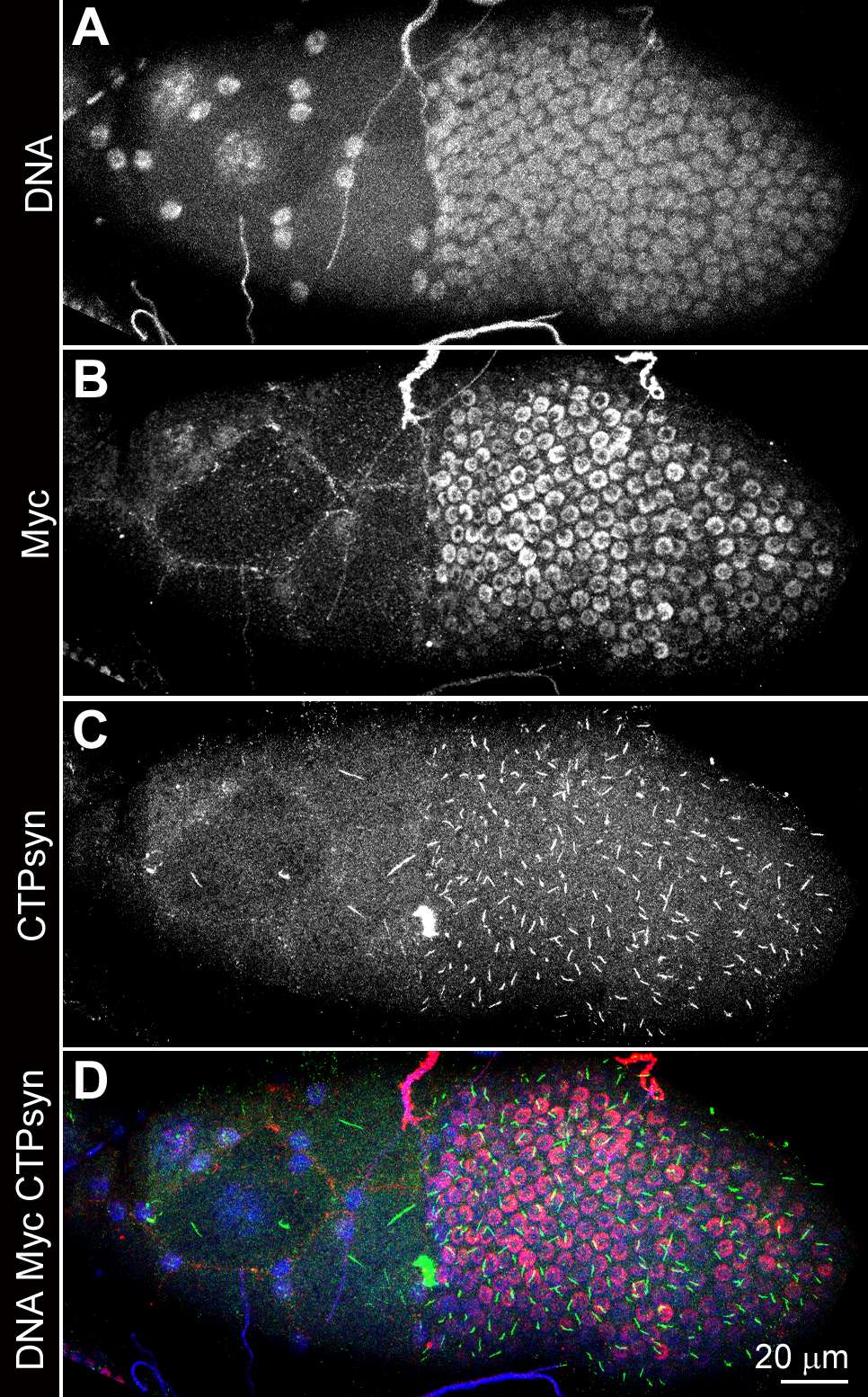

Supplement: S5 Fig — (A) DNA stained by Hoechst 33342. (B) Immunostaining with an antibody against Myc. (C) Immunostaining with an antibody against CTPsyn. (D) A merge image of A-C. Cytoophidia remain detectable in the follicle cells of stage-10a egg chamber. Myc is distributed in the nucleus of follicle cells (B). Note that the images are a surface view of the egg chamber focusing on follicle cells only. (TIF) [file pgen.1005867.s005.tif]

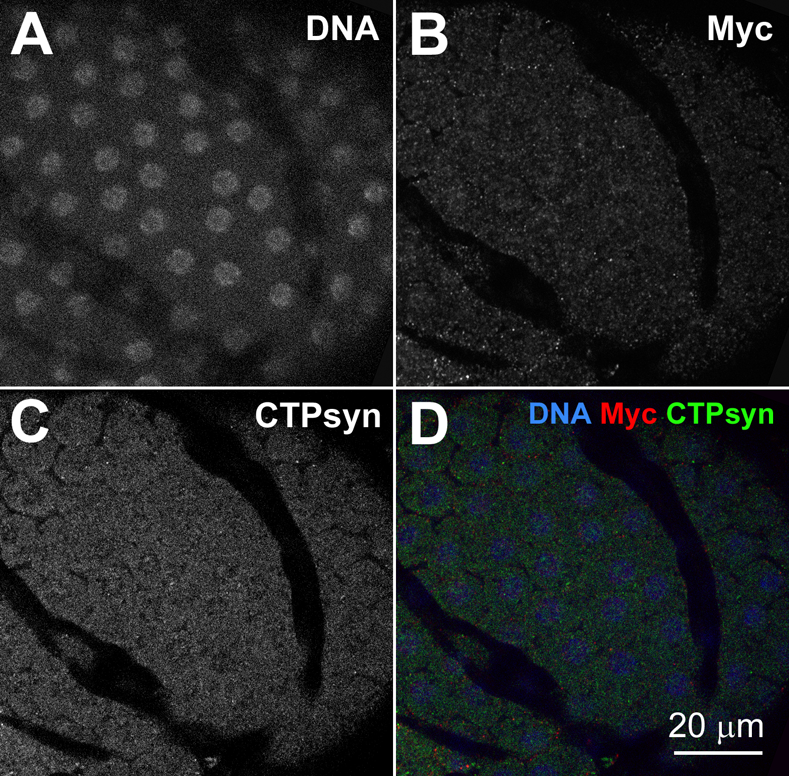

Supplement: S6 Fig — (A) DNA stained by Hoechst 33342. (B) Immunostaining with an antibody against Myc. (C) Immunostaining with an antibody against CTPsyn. (D) A merge image of A-C. In the follicle cells of stage-10b egg chamber, immunostaining of Myc shows low signal and cytoophidia are hardly detectable. Note that the images are a surface view of the egg chamber focusing on follicle cells only. (TIF) [file pgen.1005867.s006.tif]

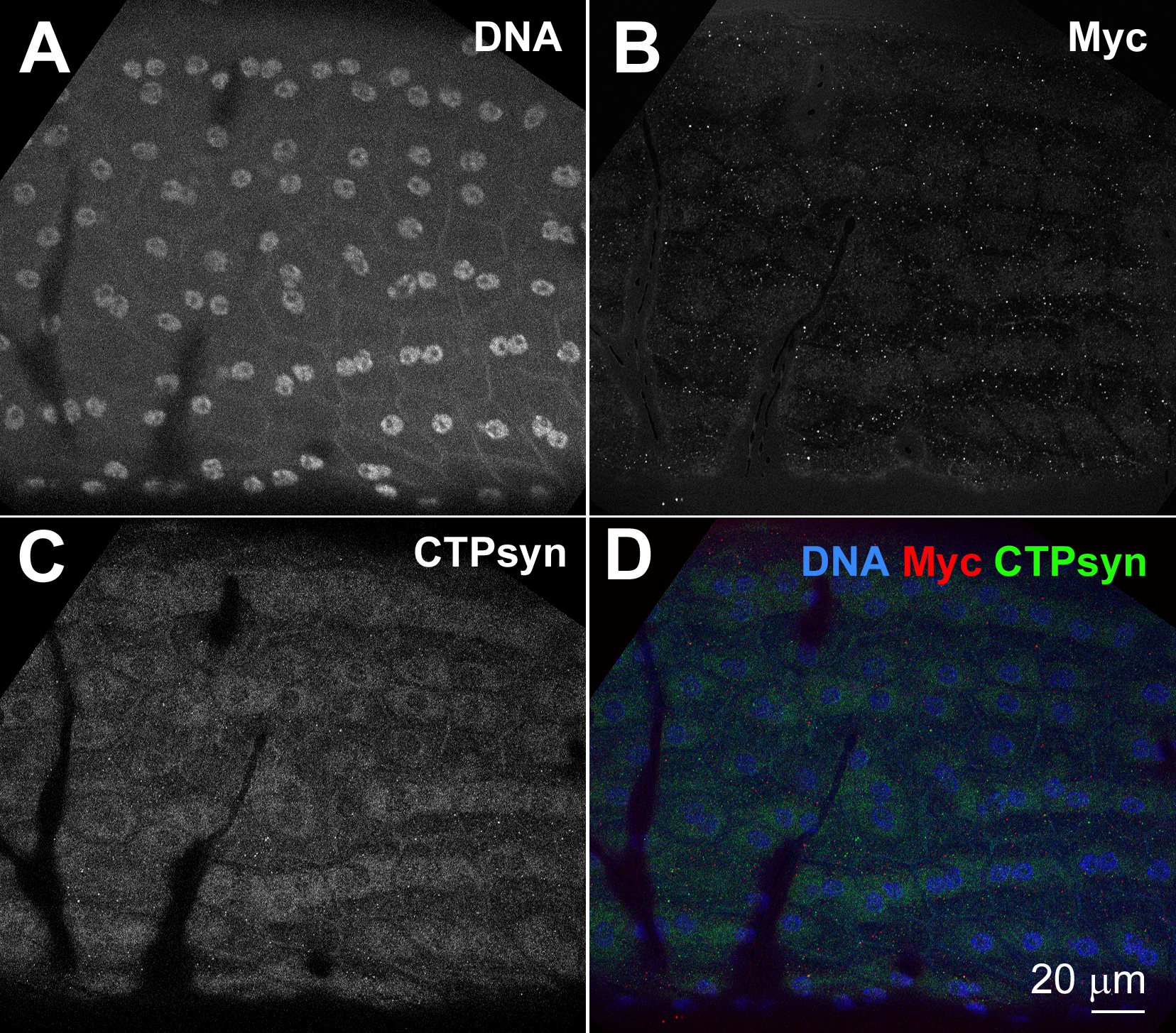

Supplement: S7 Fig — (A) DNA stained by Hoechst 33342. (B) Immunostaining with an antibody against Myc. (C) Immunostaining with an antibody against CTPsyn. (D) A merge image of A-C. In the follicle cells of stage-12 egg chamber, immunostaining of Myc shows low signal and cytoophidia are undetectable. Note that the images are a surface view of the egg chamber focusing on follicle cells only. (TIF) [file pgen.1005867.s007.tif]

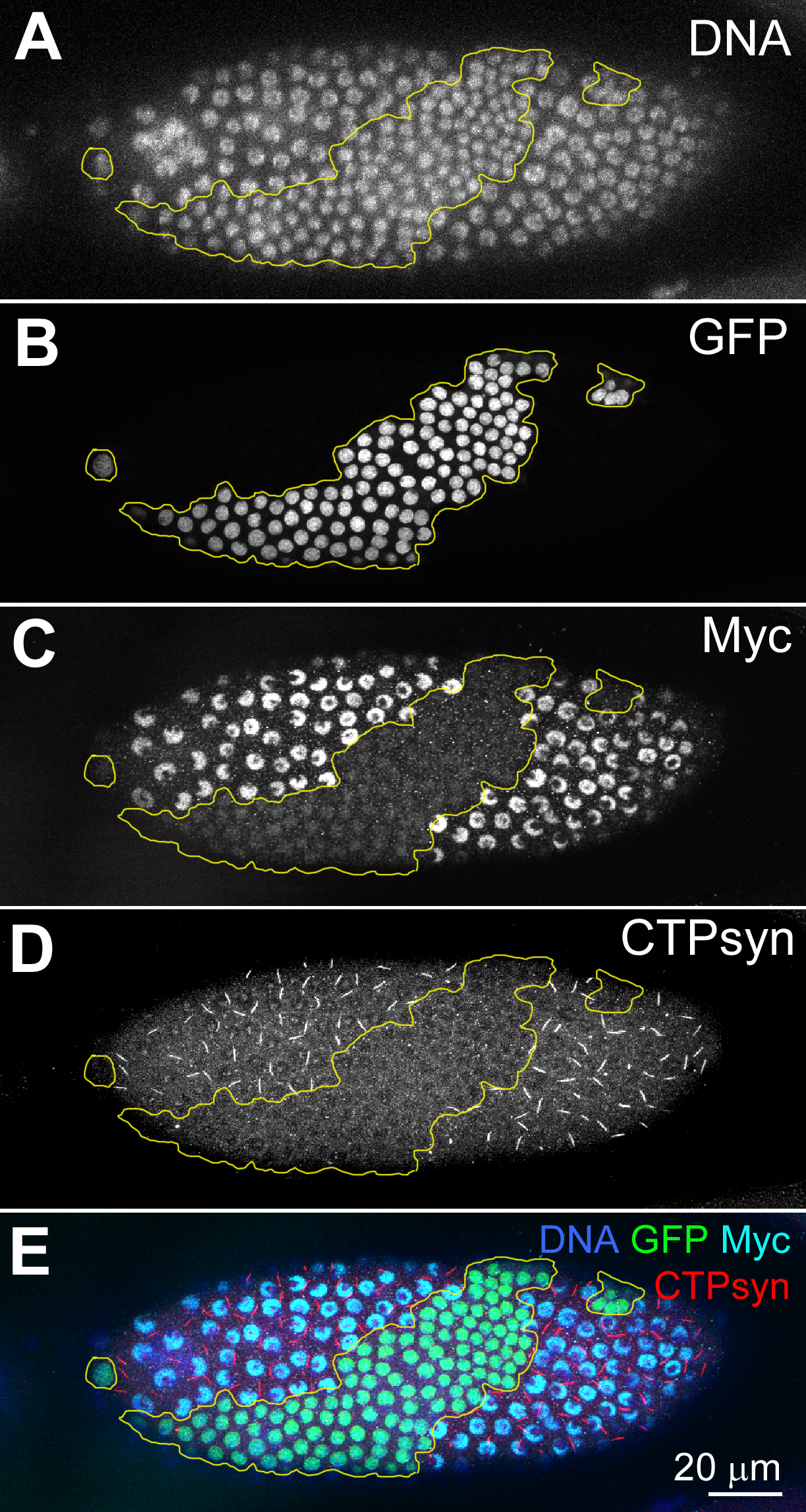

Supplement: S8 Fig — UAS-Myc-RNAiJF01762 clones marked with GFP (B, outlined in yellow in A-E) have decreased levels of Myc (C) and have no detectable cytoophidia as indicated by an antibody against CTPsyn (D). DNA staining shows that nuclei of GFP cells are smaller than those of neighbouring cells (A). (TIF) [file pgen.1005867.s008.tif]

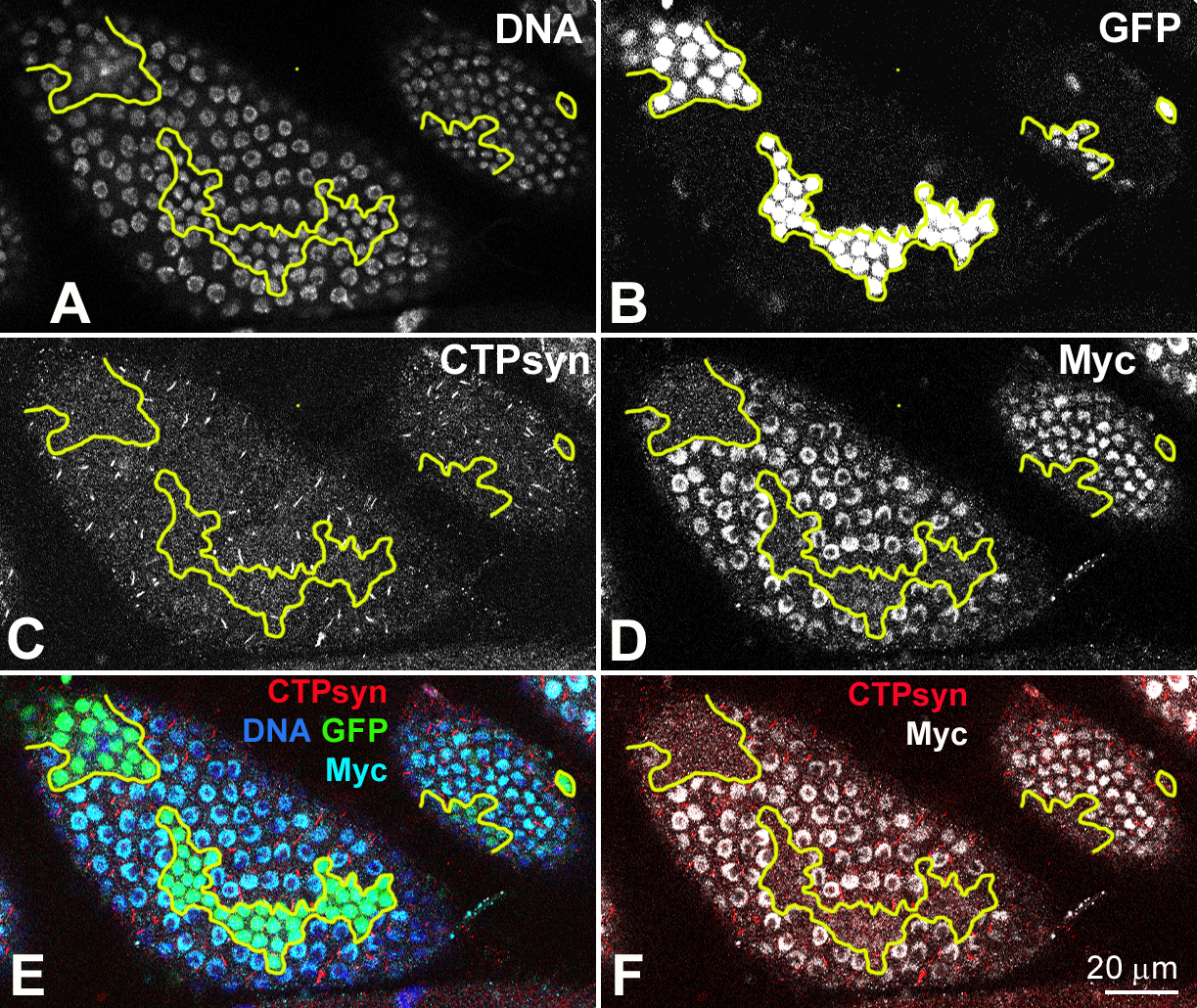

Supplement: S9 Fig — (A) DNA stained by Hoechst 33342. Characteristic small nuclear size can be seen in Myc-RNAi cells (yellow outline). (B) GFP marked cells indicating expression of Myc-RNAi (C) Immunostaining with an antibody against CTPsyn. (D) Immunostaining with an antibody against Myc. Myc levels are reduced in Myc-RNAi cells. (E) Merge of A-D. (F) Merge of C and D. (TIF) [file pgen.1005867.s009.tif]

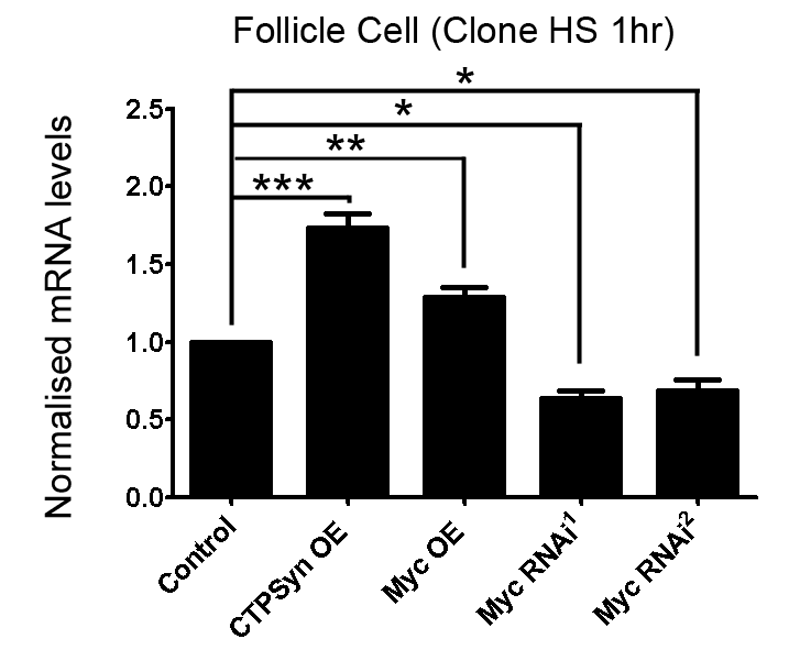

Supplement: S10 Fig — qRT-PCR was performed to detect Myc dependent changes in CTPsyn expression. The levels of CTPsyn were analysed in egg chambers overexpressing CTPsyn (UAS-CTPsyn; positive control), overexpressing Myc (UAS-Myc; to produce Myc level increases), two Myc RNAi lines (1, UAS-Myc-RNAiJF01761 and 2, UAS-Myc-RNAiJF01762, to produce Myc level decreases), and a control with no expression alterations. CTPsyn levels were seen to be positively correlated with Myc expression. Expression levels were normalised using rp49. Error bars show SEM. ANOVA was performed for significance analysis (*P<0.05, **P<0.01, ***P<0.001). (TIF) [file pgen.1005867.s010.tif]

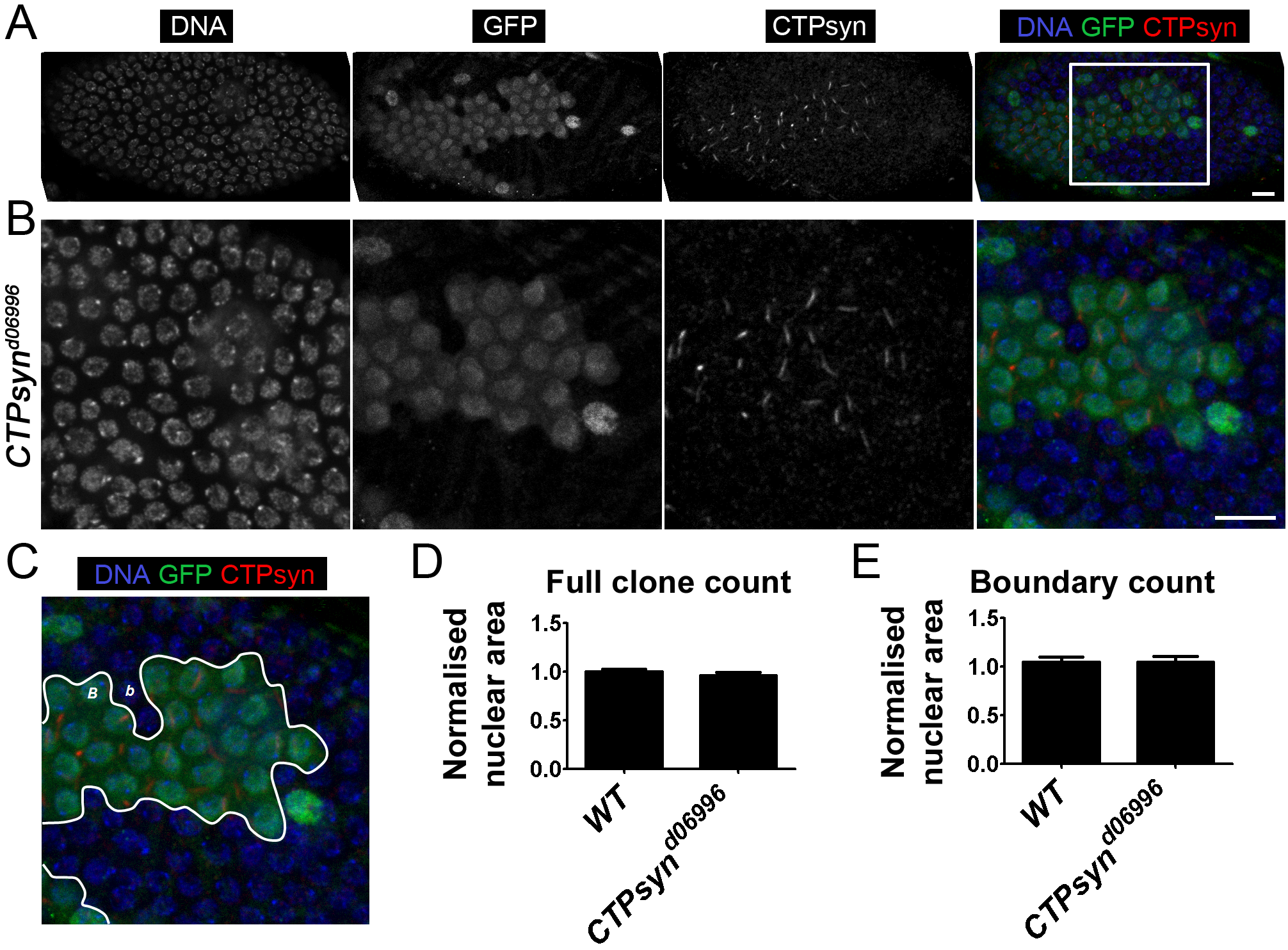

Supplement: S11 Fig — (A-B) Mitotic clones of the CTPsynd06996 mutant. B shows a zoom of the region of follicle cells depicted by the box in A, panel 4. Mutant cells, that do not express GFP, contain no observable CTPsyn filament (C-E) The nuclei area of both wild type (GFP +ive) and CTPsynd06996 mutant (GFP-ive) cells were measured. The analysis was performed throughout the wild type and mutant regions. (C, separated by the white line, quantified in D) and at the boundary of the clones (C, example wild-type boundary cell, ‘B’, and mutant cell ‘b’, boundary cells; quantified in E). (D, E) No differences were observed between wild type and CTPsynd06996 mutant cell nuclear area. Error bars show SEM. (TIF) [file pgen.1005867.s011.tif]

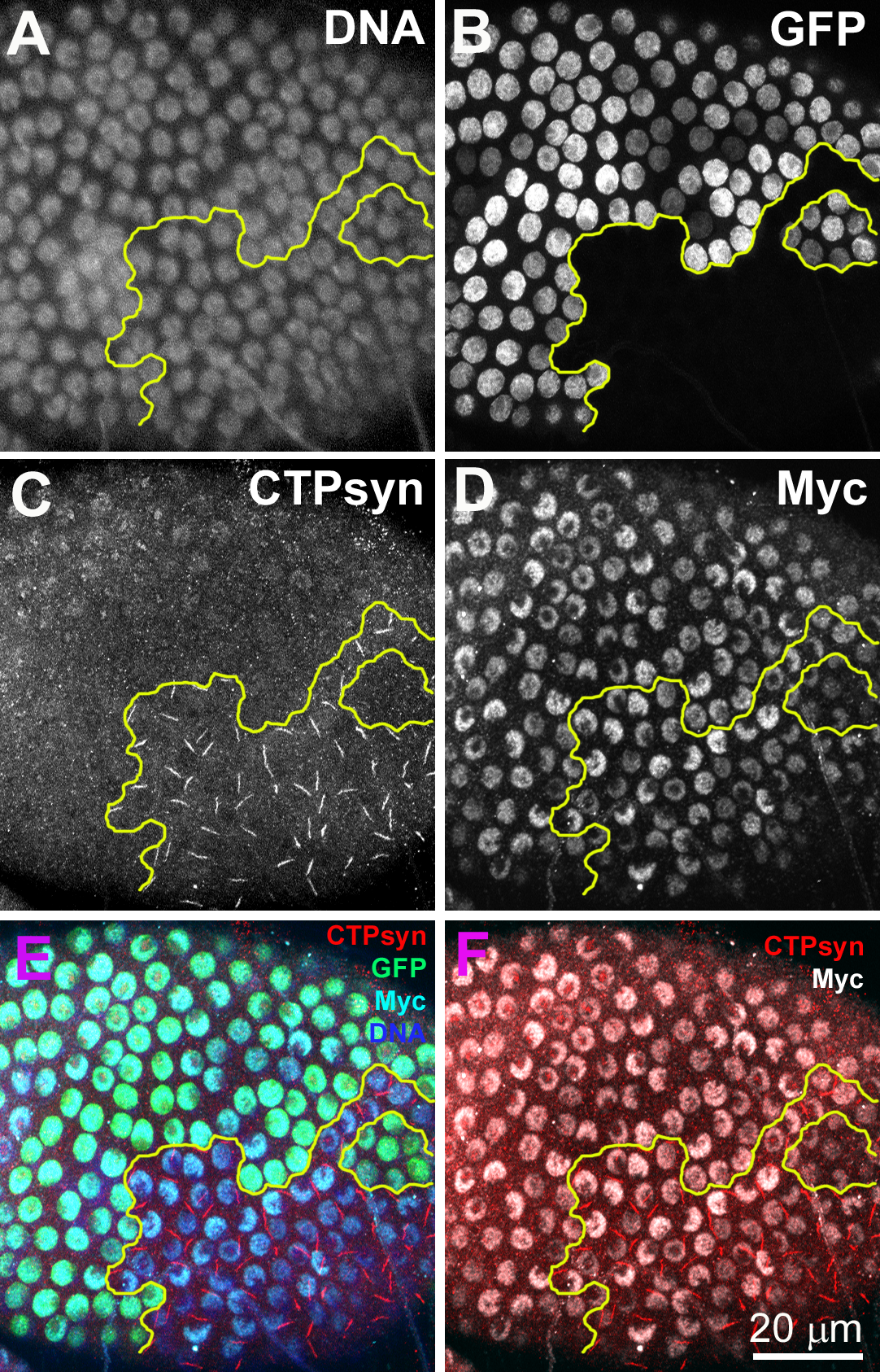

Supplement: S12 Fig — (A) DNA stained by Hoechst 33342. (B) GFP marked cells indicating CTPsyn-RNAi (yellow outline). (C) Immunostaining with an antibody against CTPsyn showing cytoophidia are not detectable in CTPsyn-RNAi cells (green cells in E). (D) Immunostaining with an antibody against Myc. Myc levels are unchanged in CTPsyn-RNAi cells, comparing with those in non-clonal cells. (E) Merge of A-D. (F) Merge of C and D. (TIF) [file pgen.1005867.s012.tif]

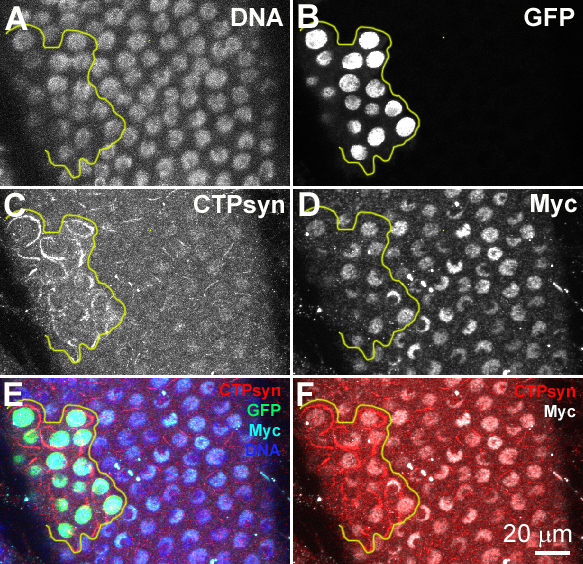

Supplement: S13 Fig — (A) DNA stained by Hoechst 33342. (B) GFP marked cells indicating CTPsyn overexpression (yellow outline). (C) Immunostaining with an antibody against CTPsyn showing cytoophidia increase in length and thickness in clonal cells (green cells in E), comparing to non-clonal cells. (D) Immunostaining with an antibody against Myc. Myc levels are unchanged in CTPsyn overexpressing cells, comparing with those in non-clonal cells. (E) Merge of A-D. (F) Merge of C and D. (TIF) [file pgen.1005867.s013.tif]
